# Obesity and Metabolic Comorbidities: Environmental Diseases?

**DOI:** 10.1155/2013/640673

**Published:** 2013-03-18

**Authors:** Carla Lubrano, Giuseppe Genovesi, Palma Specchia, Daniela Costantini, Stefania Mariani, Elisa Petrangeli, Andrea Lenzi, Lucio Gnessi

**Affiliations:** ^1^Department of Experimental Medicine, Section of Medical Pathophysiology, Endocrinology and Food Science, “Sapienza” University, Viale Regina Elena, 324, 00161 Rome, Italy; ^2^Institute of Molecular Biology and Pathology, National Council of Research (CNR), Piazzale Aldo Moro, 7, 00185 Rome, Italy

## Abstract

Obesity and metabolic comorbidities represent increasing health problems. Endocrine disrupting compounds (EDCs) are exogenous agents that change endocrine function and cause adverse health effects. Most EDCs are synthetic chemicals; some are natural food components as phytoestrogens. People are exposed to complex mixtures of chemicals throughout their lives. EDCs impact hormone-dependent metabolic systems and brain function. Laboratory and human studies provide compelling evidence that human chemical contamination can play a role in obesity epidemic. Chemical exposures may increase the risk of obesity by altering the differentiation of adipocytes. EDCs can alter methylation patterns and normal epigenetic programming in cells. Oxidative stress may be induced by many of these chemicals, and accumulating evidence indicates that it plays important roles in the etiology of chronic diseases. The individual sensitivity to chemicals is variable, depending on environment and ability to metabolize hazardous chemicals. A number of genes, especially those representing antioxidant and detoxification pathways, have potential application as biomarkers of risk assessment. The potential health effects of combined exposures make the risk assessment process more complex compared to the assessment of single chemicals. Techniques and methods need to be further developed to fill data gaps and increase the knowledge on harmful exposure combinations.

## 1. Introduction

Obesity is an increasing health problem; more than half of the European population is overweight and up to 30% is obese and its prevalence worldwide doubled since 1980 (World Health Organization 2011) [1]. Similarly, increased body weights have also been reported in pets and laboratory animals over the past decades [2]. Obesity is a condition characterized by significant clinical implications, such as comorbidities and somatic fragility, which seriously affect independence, psychological wellbeing, and overall quality of life [3, 4]. Obesity is associated with type 2 diabetes mellitus (DM), dyslipidemia, cardiovascular disease, cancer, and obstructive sleep apnea [5, 6]. Medical treatments are often ineffective and bariatric surgery is the only available therapeutic modality associated with clinically significant and relatively sustained weight loss in subjects with morbid obesity [7–9].

Proinflammatory factors are increased in obesity and DM, and the prevalent metabolic state is defined by the term “glucolipotoxicity,” in which excess extracellular glucose and fatty acids exert various damaging effects. Obesity and DM-associated oxidative stress eventually lead to systemic inflammation and endothelial cell dysfunction, central to the development of cardiovascular diseases and metabolic syndrome [10, 11].

Excess caloric consumption and a sedentary lifestyle are the only recognized risk factors for obesity and DM but alone do not account for the current worldwide obesity epidemic. New hypotheses are emerging to explain the etiopathogenesis of these conditions, including environmental chemicals, stress, immunological alterations, micronutrient deficits, and gut microbiota [12, 13]. Genetic modifications could be involved in predisposition to obesity; however the human genome has not undergone significant modifications over the last years. On the contrary, the correlation between accumulation of synthetic chemicals and increase of obesity prevalence might not be a random event. This dramatic change in the environment has led to the hypothesis that some environmental pollutants act as Endocrine Disrupting Chemicals (EDCs), interfering with various aspects of metabolism and of energy balance [14].

## 2. Endocrine and Metabolic Disruption

### 2.1. EDCs

EDCs have been defined as exogenous substances that alter function(s) of the endocrine system and consequently cause adverse health effects in an intact organism, or its progeny, or (sub)populations. (International Programme for Chemical Safety—IPCS). EDCs can mimic the action of natural hormones or interfere with their production, release, metabolism, and elimination [15]. These substances may derive from natural, animal, human, or plant sources. Phytoestrogens are EDCs found naturally in certain plants, including foods like whole grains, leafy greens, beans, and garlic, and can mimic the action of estrogen, showing some beneficial effect on bone mineral density, insulin resistance, and cardiovascular risk factors in women after menopause [16–20]. Another class of EDCs belongs to the group of heavy metals (i.e., cadmium, mercury, and arsenic). These metals may cause occupational or residential exposure [21]. Heavy metal toxicity can result in reduced mental and central nervous function and damage to blood composition, lungs, kidneys, liver, breast, and other vital organs [22, 23]. Allergies are not uncommon and repeated long-term exposure with some metals may even cause cancer [24, 25]. Major concerns, however, are currently focused on industrial products. In fact, most EDCs are synthetic chemicals designed for use in a variety of industries. Classes of these chemicals are solvents or lubricants and their byproducts (polychlorinated biphenyls (PCBs) and dioxins), plastics, plasticizers (bisphenol A (BPA)), phthalates; pesticides (methoxychlor, chlorpyrifos, DDT) fungicides (vinclozolin), herbicides (atrazine), and antibacterials (triclosan). There are several reports of pollution from PCBs and dioxins that show a direct causal relationship between a chemical and the manifestation of an endocrine, immunological, or metabolic dysfunction in humans and in wildlife [26–29] but the more common event is the widespread persistent exposure to a broad mix of chemicals.  Table 1 shows a brief summary of the principal incidents caused by dioxins contamination in the last fifty years.

### 2.2. Mechanisms of Action

Some EDCs were designed to have long half-lives and therefore are persistent contaminants, do not decay easily, may not be metabolized, or may be broken down into more toxic compounds [30]. Others, such as BPA, although not very persistent in the environment, are so widespread in their use that there is a prevalent human exposure [31]. Humans and wildlife are exposed daily to a variety of compounds, and it is thus likely that even if none reach an effective level, the combination or mixture of chemicals may become dangerous. These chemical mixtures enter the food chain and accumulate in animals up to humans. Exposure occurs also through drinking contaminated water, breathing contaminated air, or contacting contaminated surfaces. They may exert nontraditional dose-response curves, the so-called U-shaped or inverted U-shaped dose-response curve. As a consequence, any level of exposure may cause endocrine or metabolic abnormalities, particularly if the exposure occurs during a critical developmental period, and low doses may even be more potent than high doses [32, 33]. The age of exposure is important [34], since the environment to which a developing organism (fetal life, childhood) is exposed interacts with the individual's genes to determine the propensity to develop a disease later in life. The majority of environmental factors and toxicants do not alter DNA sequence or promote genetic mutations. Therefore, they may promote abnormal phenotypes or disease through modifications of factors that regulate gene expression such as DNA methylation and histone acetylation [35–39]. 

EDCs can bind and activate multiple hormone Nuclear Receptors (NRs). Various EDCs share receptors, and thus additive or even synergistic effects may be observed [40]. Among NRs, Estrogen Receptors (ERs) regulate many aspects of metabolism, including glucose transport, glycolysis, mitochondrial activity, and lipid metabolism [41]. It is likely that ER activation modulates neural networks controlling food intake and adipose tissue [42, 43]. Male and female ER knock-out mutant mice show increased insulin resistance and impaired glucose tolerance [44]. Neonatal exposure to a low dose of the estrogenic drug diethylstilbestrol (DES) stimulated a subsequent increase in body weight and an increase in body fat in mice [45]. BPA, a breakdown product of coatings in food and beverage containers, may act as an ER agonist. In the US population, exposure is nearly ubiquitous, and BPA has been detected in fat, blood, and urine [46]. Short exposure to BPA provokes chronic hyperinsulinemia, with perturbations of glucose and insulin tolerance tests [47]. Furthermore, high- or low-dose exposure to BPA during gestation up to puberty leads to hyperlipidemia with increased body and adipose tissue weight in both sexes [48, 49]. BPA exposure has been shown to disrupt multiple metabolic mechanisms, suggesting that it may contribute to obesity in humans [50–52]. Other studies have demonstrated associations between urinary BPA concentration and adult DM, cardiovascular diseases, obesity, and abnormalities in liver function [53, 54] A longitudinal study of apparently healthy adults showed an association between baseline urinary BPA concentration and later-life coronary artery disease [55]. 

EDCs may also modulate other hormone NRs, particularly thyroid hormone receptor (TR) and glucocorticoid receptor (GR). BPA acts as a TR antagonist in vitro, increases serum thyroxin, and alters RC3/neurogranin expression in the developing rat brain [56]. Brominated Flame Retardants (BFRs) also disrupt the TR pathway, and exposure of rats to Polybrominated Diphenyl Ethers (PBDEs) resulted in a significant increase in lipolysis and a significant decrease in glucose oxidation [57]. Organotins and PCBs can bind GR and alter 11beta-hydroxysteroid dehydrogenase type 2 activity [58]. 

The body is protected from the accumulation of toxic chemicals by the expression of drug-metabolizing enzymes and transporters. This adaptive response incorporates at least three NRs: pregnane X receptor (PXR), constitutive androstane receptor (CAR), and aryl hydrocarbon receptor (AhR), as well as xenobiotic metabolic and transporter systems. PXR and CAR are members of the NR super family of sensor receptors and contribute to fatty acid, lipid, and glucose metabolism, and CAR seems to be an anti-obesity NR that ameliorates DM and fatty liver accumulation [59–61]. Endogenous ligands of PXR and CAR include some bile acid derivatives, pregnanes, androstane metabolites, and other metabolic products of steroids; exogenous compounds include herbal medicines pharmaceutical drugs and synthetic steroid hormones. A number of EDCs activate both PXR and CAR: nonylphenol, Di (2-Ethylhexyl) Phthalate (DEHP), Mono-(2-Ethylhexyl) Phthalate (MEHP), BPA, some PCBs perfluorooctane sulfonate (PFOS), perfluorooctanoic acid (PFOA), and the organochlorine methoxychlor [62]. AhR is a xenosensor that mediates the biological response to a wide spectrum of xenobiotics; in particular, AhR mediates the toxic effects of dioxins [63]. Endogenous molecules that bind AhR are lipoxin 4, leukotriene derivatives, biliverdin, and bilirubin. Xenobiotics that activate AhR include various dietary phytochemicals, some PCBs, and 2,3,7,8-Tetrachlorodibenzo-p-Dioxin (TCDD). The mechanisms through which AhR regulates energy metabolism are not clearly established; however, crosstalk with ER may be involved. In addition, AhR also indirectly affects adipogenesis through inhibition of Peroxisomal Proliferator-Activated Receptor (PPAR)*γ* expression [64]. 

### 2.3. Health Effects

All major endocrine organs are vulnerable to endocrine disruption, including the Hypothalamus-Pituitary-Adrenal axis, reproductive organs, the pancreas, and the thyroid gland [65, 66]. For example, it has been reported that EDCs, and estrogen mimicking agents among them, prolong the proliferation phase of immature Leydig cells, prior to their maturation [67], and can interfere with peritubular myoid cells [68], and it is known that estrogens are associated with Leydig cell tumorigenesis in mice [69–71]. The declining level of androgen during aging, associated with an increasing level of estrogen, has been hypothesized to be important in the development of benign prostatic hyperplasia [72–74], and oral exposure to low-dose BPA seems to aggravate testosterone-induced benign hyperplasia prostate in rats [75]. Several studies have suggested that estrogen exposure may increase the risk of prostate cancer [76], and various hormones such as androgens and gonadotropin-releasing hormone may play a role in prostate cancer cell growth [77, 78]. BPA and dibutyl phthalate (DBP) seem to be able to stimulate the growth of prostate cancer cells [79]. Thyroid carcinoma is the most common endocrine malignancy being about 3 times more common in women than in man [80]. Analogously, in differentiated thyroid cancer (DTC), EDCs with estrogen-like activity may be suspected to play a role in disease progression. Experimental evidence demonstrated that estrogen, but not testosterone, promotes DTC cell proliferation and that this effect could be attenuated by tamoxifen [81, 82].

In 2006, Bruce Blumberg developed the “obesogen hypothesis”, to explain the weight gain effects of certain chemicals. This hypothesis is supported by laboratory and animal research as well as epidemiological studies that shown that a variety of EDCs can influence adipogenesis and obesity [83–86]. EDCs are also known to impact hormone-dependent metabolic systems and brain functionandcan be easily found in human blood and urine and epidemiological literature on associations between EDC exposure and body weightisincreasing [87–89].

These substances target various endocrine axes and affect adipocyte physiology and more generally the regulation of energy homeostasis [90]. Several persistent organochlorine pesticides and fungicides have been implicated in obesity [91, 92]. Metabolic alterations like metabolic syndrome and type 2 DM are recognizedasobesity comorbid conditions, and EDCs exposure may be involved in their pathogenesis. Such contribution was revealed by comprehensive studies of large and well-characterized cohorts, such as the cohort used for the NHANES (National Health and Nutrition Examination Survey) project [93, 94].

Epidemiological studies show an association between dioxins exposure and type 2 DM [95]. High and low doses of dioxins affect genes linked with hepatic circadian rhythm, cholesterol biosynthesis, fatty acid synthesis, glucose metabolism, and adipocyte differentiation, in an AhR-dependent manner [96, 97]. Weight control and intermediate metabolism require a precise balance between energy input, storage, and consumption; several NRs are involved. The PPARs act as lipid sensors that act in different organs to adapt gene expression to a given metabolic status [98]. Plasticizers, surfactants, pesticides, and dioxins can modulate PPARs activity, and the phthalates are a group of well-characterized peroxisome proliferators. Mono-(2-Ethylhexyl) Phthalate (MEHP) is proadipogenic in a cell culture model, suggesting that it may act as a metabolic disruptor and may promote obesity in vivo [99, 100]. Kanayama et al. [101] showed that among 40 EDCs, organotins such as tributyltin (TBT) and bis (triphenyltin) oxide (TPTO) are activators of human PPAR*γ* [102]. 

## 3. Oxidative Stress and Mitochondrial Dysfunction

An increase in oxidative stress-associated inflammation has been hypothesized to be a major mechanism in the pathogenesis of obesity-related diseases. Additionally, a rise in inflammatory cytokine levels might drive a further increase in oxidative stress, setting up a vicious cycle [104, 105]. When perturbed, the mitochondrial system alters the output of matter and energy and this may result in a pathological phenotype, such as that of obesity, dyslipidemia, metabolic syndrome, hypertension, and cancers. The failure of the skeletal muscle mitochondria to oxidize fat properly leads to ectopic lipid deposits. Cellular infiltration by excess triglycerides can impair cellular function and can also lead to oxidative stress through increased ceramide formation, increased lipid peroxidation, inflammatory cytokine production, and excess Reactive Oxygen Species (ROS) formation [106, 107]. When ROS production is increased, the disturbed balance results in a prooxidative condition. This oxidative stress can then damage various cellular structures and triggers an inflammatory response associated with adiposity, insulin resistance, and metabolic syndrome, suggesting that oxidative stress could be an early event in the pathology of these chronic diseases. [108, 109]. Recently, much evidence has emerged showing that environmental toxins, including Persistent Organic Pollutants (POPs), can affect mitochondrial function and subsequent insulin resistance. In this regard, many herbicides, insecticides, rodenticides, industrial products, and industrial toxic wastes might affect mitochondrial function and cause pro-oxidative conditions [110–112].

## 4. Exposure Monitoring

Various molecules, involved in antioxidant and detoxification pathways, have potential application as biomarkers in biomonitoring and risk assessment. 

The National Academy of Science, in 1987, defined a biomarker as “a xenobiotically induced variation in cellular or biochemical components or processes, structures, or function that is measurable in a biological system (body fluids, cells, or tissues)” [113]. Biomarker responses in fish are routinely used to assess exposure of anthropogenic chemicals in the aquatic environment. The use of biomarkers could complement the current methods used to determine the presence of environmental pollutants and might also help to predict human health risks [114]. Among the various types of biomarkers in ecotoxicological studies are the following: cytochrome P450 activity (an indicator of the exposure and effect of organic contaminants, such as polycyclic aromatic hydrocarbons (PAHs), PCBs, and pesticides), the inhibition of Acetyl cholinesterase (AChE) activity (a biomarker of the exposure and effect of organophosphate (OP) and carbamate (CAR), metallothionein synthesis in hepatic and other tissues (exposure to the metals Zn, Cu, Cd, Hg, and Fe and some pesticides), antioxidant enzymes such as superoxide dismutase, catalase, and glutathione transferase (exposure to ROS, free radicals, and pollutants causing oxidative stress and lipid peroxidation, such as pesticides and metals), and vitellogenin induction (estrogenic substances) [115–121]. Recently, redox markers have been used to biologically define Multiple Chemical Sensitivity (MCS) [122]. The development and use of biomarkers in ecotoxicology for providing sensitive early warning signals of incipient ecological damage is motivated by the inherent instabilities of many EDCs and the chemical specificity of some biomarkers on underlying mechanisms of toxic action. However, little is known about how cocktail effects affect these biomarker responses, and chemical safety-levels are traditionally based on experiences from lab studies with single chemicals, which are unfortunate as a chemical can be more toxic when it is mixed with other chemicals, because of the cocktail effect, for example, if there is a risk for increased bioavailability of certain pollutants that can result in harmful bioaccumulation or if there is an increased risk for accumulation of toxic metabolites or if there is an increased risk for depletion of endogenous hormones. The possible involvement of receptor crosstalks, inhibition, or activation on key biotransformation enzyme and transporter proteins such and multidrug resistance-associated proteins needs to be addressed for estimation of possible adverse pharmacokinetic interactions [123].

## 5. Concluding Remarks

EDCs clearly contribute to diverse male and female human health problems such as decreased male sperm counts, increased incidence of hypospadias and cryptorchidism, altered male: female birth sex ratios, decreased fertility, and increased incidence of breast and testicular cancers and may be responsible for neurodevelopmental deficits in children. Recently, human exposure to EDCs has been associated with the development of some of the main diseases of the industrialized world, particularly metabolic disorders like obesity, diabetes, and metabolic syndrome. POPs such as organochlorine pesticides, dioxins, and polyfluoroalkyl compounds and no persistent pollutants such as BPA and several phthalates are endowed with metabolic disruption activity. The European Union has sponsored several international research project to investigate various obesity-related effects from EDCs exposure (Table 2), regarding the diverse types of chemical compounds involved, the influence of climate changes on contaminant spreading, the age of exposition (developmental basis of adult diseases), food safety, and human bio monitoring. A greatest challenge in environmental toxicology is to understand effects of mixture toxicity (cocktail effects) in humans and in wildlife. Considering that metabolic perturbations are only one small aspect of the EDCs-related problems to be solved and that we know only the tip of the iceberg, new integrative approaches are required to understand the complexity of the cocktail effect and its consequences when exposure occurs at various life stages [124]. Procedures for risk assessment of chemical mixtures, combined, and cumulative exposures are under development, but the scientific database needs considerable expansion [114]. In particular, there is a lack of knowledge on how to monitor effects of complex exposures. As described here, solid evidence shows that endocrine disrupters can interact and even produce synergistic effects. They may act during sensitive time windows and biomonitoring their effects in epidemiological studies is a challenging task. The potential health effects of combined exposures make the risk assessment process more complex compared to the assessment of single chemicals. Techniques and methods need to be further developed to fill data gaps and increase the knowledge on harmful exposure combinations.

## Figures and Tables

**Table 1:** Examples of dioxin contamination incidents.

Year	Country	Type of accident	Cause
1960–1975	Vietnam	Contamination by a defoliant, agent orange	Contaminated agent orange
1976	Italy (Seveso)	Contamination by a cloud of toxic chemicals of an area of 15 square kilometers	Accident at a chemical factory
1998	Germany	Contamination of milk	Contaminated citrus pulp pellet from Brazil
1999	Belgium	Contamination of poultry and eggs	Animal feed contaminated with illegally disposed PCB-based waste industrial oil
2004	The Netherlands	Contamination of milk	Contaminated clay in animal feed
2007	Europe	Contamination of guar gum (food additive)	Guar gum from India contaminated by pentachlorophenol that contains dioxins as further contamination
2008	Ireland	Contamination of pork meat and products	Contaminated animal feed

**Table 2:** Examples of ongoing European research projects on endocrine disrupters [103].

Project acronym and duration	Project title	Research team	Focus
CONFFIDENCE (2008–2012)	Contaminants in food and feed: inexpensive detection for control of exposure	Participating laboratories: 17 (NL, CZ, ES, DE, DK, BE, UK, IT, FI, CH)	The main aim is to further improve food safety in Europe by the development of faster and more cost-efficient methods for the detection of a wide range of chemical contaminants (persistent organic compounds, perfluorinated compounds, and heavy metals) in different food and feed commodities
ARCRISK (2009–2013)	Arctic health risks: Impacts on health in the Arctic and Europe owing to climate-induced changes in contaminant cycling	Participating laboratories: 22 (NO, SE, DK, FI, DE, UK, ES, SI, CH, CZ, RU, CA)	The main aim will be to study the influence of climate change on contaminant spreading and transfer and the resultant risk to human populations in the Arctic and other areas of Europe
COPHES (2009–2012)	European coordination action on human biomonitoring	Participating laboratories: 35 (BE, DE, ES, UK, FR, DK, IT, EE, SI, NL, AT, RO, LT, HR, EL, CY, PT, SE, NO, HU, CH, SK, PL, CZ, IE, FI, LU)	The main goal is to develop a coherent approach to human biomonitoring in Europe, addressing the aims of Action 3 of the European Environment and Health Action Plan.
ENFIRO (2009–2012)	Life cycle assessment of environment-compatible flame retardants (prototypical case study)	Participating laboratories: 12 (NL, UK, SE, DE, IT)	ENFIRO will offer a prototypical case study on substitution options for BFRs resulting in a comprehensive dataset on viability of production and application, environmental safety, and a complete life cycle assessment
OBELIX (2009–2013)	Obesogenic endocrine disrupting chemicals: linking prenatal exposure to the development of obesity later in life	Participating laboratories: 7 (NL, BE, NO, FR, SK)	Examination of the hypothesis that prenatal exposure to endocrine disrupting compounds in food plays a role in the development of obesity later in life
